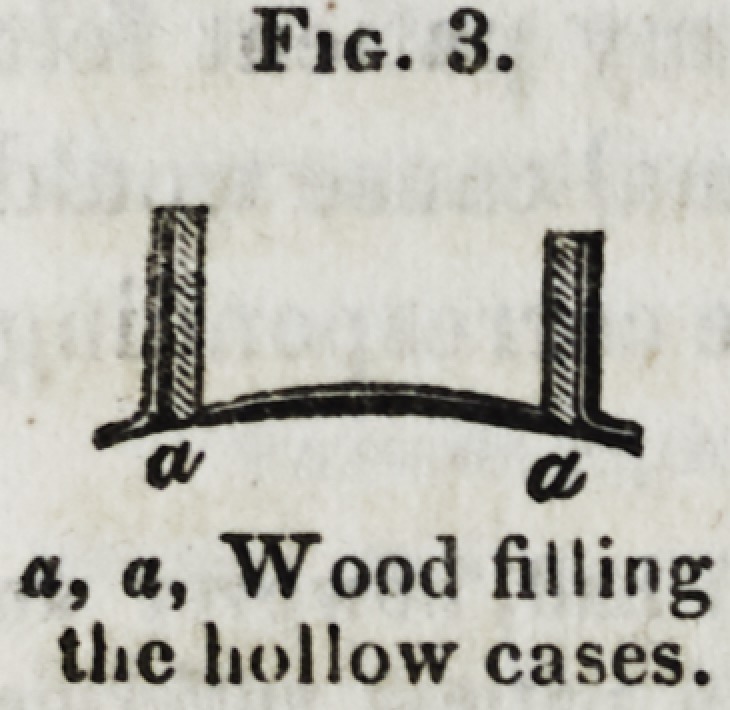# New Method of Supporting Artificial Teeth

**Published:** 1854-04

**Authors:** 


### ARTICLE VIII.

### *New Method of Supporting Artificial Teeth.*

GENTLEMEN:

In the "American Journal of Dental Science," for October, 1853, there appears an article, by Dr. Wm. M. Hunter, of Cincinnati, Ohio, entitled, "another European principle Americanized;" intended by its author to illustrate a new method of maintaining gold plates *in situ*, by the use of gold tubes and compressed wood—a method of indisputable value, and applicable as a very efficient mode of supporting artificial teeth, in the majority of cases, not only with firmness, but with great comfort also to the patient.

We, on this side of the Atlantic, are considerably indebted to our American brethren for many signal improvements, both in mechanical dentistry and in the surgical application of the science; nevertheless, a feeling of honest pride induces me to assert my claim to whatever merit is due me, as the originator of the method described by your esteemed contributor.

In the "Medical Times and Gazette" of January 31st, 1852, an article appeared under the title of "remarks on a new method of supporting artificial teeth; by C. Stokes, Esq., M. R. C. S." This article, which I wrote, was intended to explain a method of applying wood, in combination with gold tubes, as a means of supporting gold frames in the mouth. The use of wooden pegs, or cylinders of hard wood in bone pieces, was very generally known and applied; but I can assert most positively that until the article which I wrote on the subject appeared in the pages of the periodical alluded to, the application of the principle of gold tubes and compressed wood had never been made either in England or on the Continent.

I acknowledge, with considerable gratification, Dr. Hunter's direct testimony in favor of the system I have brought forward and advocated. In this country, it has received the approbation of some of the most scientific, as well as practical, dentists of the day, and I can affirm, with perfect truth, that increased experience, extending now over a period of nearly three years, has fully confirmed my previous conviction of the value of the principle, and I am quite confident that the day is not far distant, when the use of rigid gold bands, with their many concomitant disadvantages, will be reckoned as one of the unscientific appliances of a by-gone age.

I am, gentlemen, your obedient humble servant,

CHARLES STOKES, M. R. C. S.

65 *Brook street, Hanover Square, London, January 9th,* 1854.

The use of artificial teeth is, at the present time, so universal in every rank of life, and we may add, their utility is so indisputable, in heightening the charms of personal appearance, and in assisting to maintain the general health in a favorable condition, that every suggestion for their practical improvement is necessarily invested with considerable interest, and can hardly fail to engage the attention of every class of society, particularly that of the intelligent medical practitioner, to whose judgment and opinion in this special branch of practice reference is constantly made by the public. If in the use of artificial teeth there are admitted advantages, on the other hand, unfortunately, the benefit is materially lessened by the injurious action which these substitutes exercise on the natural teeth; especially when bands or clasps are used as the means of supporting them.

When we examine carefully the enamel of teeth on which bands have rested for any length of time, we find, in the greater number of cases, that its structure has undergone a considerable change; the carbonates and phosphates are decomposed, and partially dissolved, leaving the surface of the enamel porous, and of a chalky appearance; the dentine is, under these circumstances, soon affected, the tubes break down, and caries in its true form is established. The system of maintaining artificial teeth *in situ* by atmospheric pressure, is not open to this objection; and in cases where the gums and palate are favorably formed, is a successful method of supporting them without inconvenience, and with sufficient firmness to assist mastication very materially. The proximate cause of the loss of teeth under the circumstances already mentioned, is undoubtedly the lodgment, between the bands and teeth to which they are attached, of particles of food, which, in combination with the saliva, undergo chemical changes, whence an acid is generated. If we remove from the bands of artificial teeth, a portion of this pulpy mass, which is found, in greater or less quantities, lying between them and the necks of the teeth round which they pass, and apply it to litmus paper, the latter becomes instantly reddened. It is the perpetual contact of this acid matter with the teeth, that gradually but surely effects such changes in their structure as ultimately to involve their loss. The true character of this acid product is at present undetermined by chemists of great eminence;* a fact which is of minor importance, per-

* "Various authors have assumed that lactates of the alkalies are present in normal saliva, and have referred the acid re-action which is occasionally noticed in that fluid to the presence of free lactic acid; but in the small amount of solid residue which is left by the saliva, I have never been able to establish with certainty the presence of lactates, even when operating upon considerable haps, compared with its destructive action on the teeth under circumstances favorable to its development. A question of greater value undoubtedly is, by what means can this evil be obviated? or, if not entirely obviated, at all events materially lessened.

I propose to lay before the profession a system of mechanical aid in supporting artificial frames, which I have found practically to effect this object. A gentleman consulted me in the early part of the past year, in consequence of severe attacks of pain which he experienced in an upper molar tooth. He had worn, for nearly two years, artificial teeth, carved from the dentine of the hippopotamus, and adapted to fill certain spaces in the upper jaw, of which the following diagram, (*Fig.* 1,) is an accurate representation.

FIG. 1.

On examining the right molar, I found the neck extensively affected by caries, which was, in my opinion, the consequence of the re-action of the acidulated pulp to which I have already alluded. Some preliminary treatment enabled me to subdue the pain, and eventually to plug the tooth. As my patient felt assured that the continued operation of the original cause would again induce caries, either in that tooth or in the corresponding quantities, obtained both from man and from the horse. I had, however an opportunity of collecting large quantities of the saliva of a patient suffering under *diabetes mellitus*, and in this case I convinced myself beyond all doubt of the presence of free lactic acid."—*Vide Lehmann's Physiological Chemistry*, p. 94.

molar on the left side, both of which were employed to support the bone frame, he was very desirous of having such a system of artificial teeth adapted to his mouth as would avoid this contingency for the future. After some consideration, I devised a plan by which I felt assured that his wishes might be fulfilled, not only without injury to his remaining teeth, but with an increase of security, and also of utility.

I substituted a gold plate for the inconvenient mass of dentine which he had worn, and proposed to support the new frame and its accompanying teeth by means of elastic wood, enclosed in a flattened hollow case, accurately adjusted to the side of each molar tooth in the upper jaw, so as to secure contact at every point in a line drawn from the neck to the crown of the tooth; the position of these hollow cases on the plate is seen in the accompanying *Figure* (2.)

FIG. 2.

Gold plate with teeth mounted on it, adapted to fill the spaces shown in *Fig.* 1. *a, a*, hollow cases to receive the wood.

A sectional view of these cases, with the wood inserted, is given in *Fig.* 3. I inserted into these cases compressed hickory-wood, which, by increasing slightly in volume when moistened by the fluids of the mouth, pressed the sides of the molars with so much firmness and uniformity as to secure the accurate adaptation of the frame to the gums, with a completeness which satisfied every requirement. I have, since the period to which I have referred, applied the same principle

FIG. 3.

*a, a*, Wood filling the hollow cases.

in numerous cases of partially edentulous jaws, varying its application according to the particular character presented by each case; in all of which the result has been highly satisfactory. I have therefore no hesitation in recommending its adoption in every instance in which it is practicable, being very confident that it combines security with great facility of removal and replacement; and, by preventing the lodgment of particles of food liable to undergo chemical changes, we necessarily remove an exciting cause, which, in many instances, involves the destruction of healthy teeth; in addition to which, we effectually banish from the mouth that unpleasant odor which so frequently accompanies the use of artificial teeth constructed in the ordinary manner.

### ARTICLE IX.

*Cleansing the Teeth.* By T. D. THOMPSON, Providence, R. I.

## Figures and Tables

**Fig. 1. f1:**
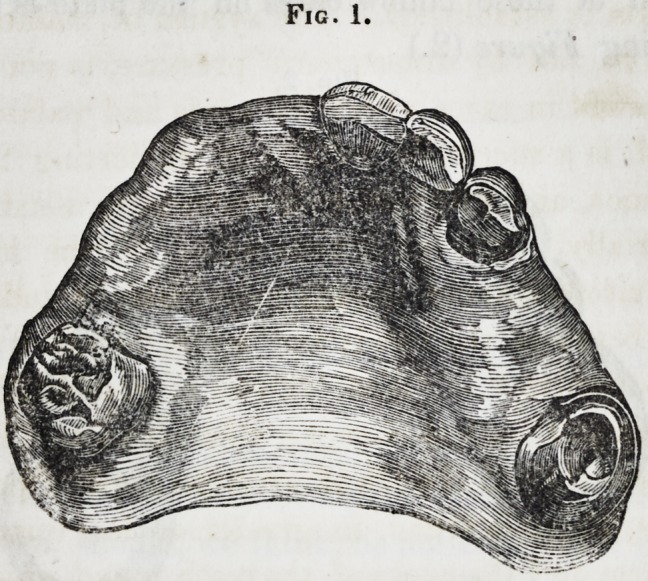


**Fig. 2. f2:**
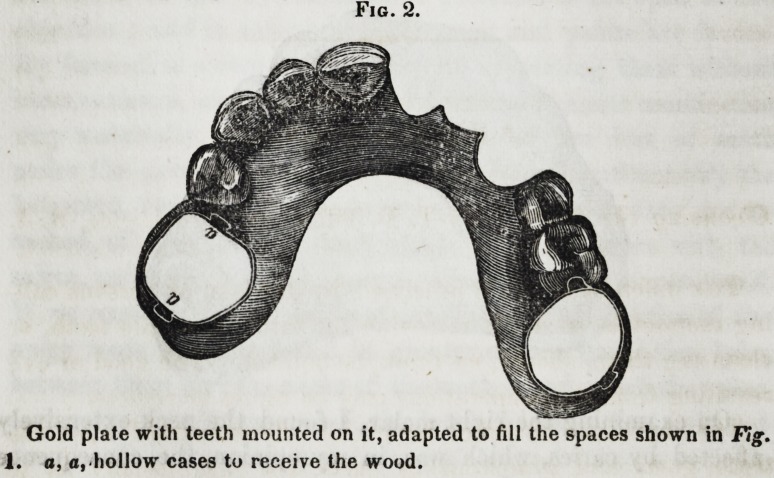


**Fig. 3. f3:**